# Management of hepatic epithelioid haemangio-endothelioma in children: what option?

**DOI:** 10.1038/sj.bjc.6601720

**Published:** 2004-03-23

**Authors:** K Sharif, M English, P Ramani, D Alberti, J-B Otte, P McKiernan, S Gosseye, M Jenney, J de Ville de Goyet

**Affiliations:** 1 Liver Unit and Department of Paediatric Oncology, Birmingham Children's Hospital, Steelhouse Lane, Birmingham B4 6NH, UK; 2 Department of Pathology, Birmingham Children's Hospital, Steelhouse Lane, Birmingham B4 6NH, UK; 3 Liver Transplant Centre, Ospedali Riuniti, Largo Barozzi 1, 24100 Bergamo, Italy; 4 Department of Paediatric Surgery, St Luc University Clinics Brussels, Avenue Hippocrate 10, B-1200 Brussels, Belgium; 5 Department of Pathology, St Luc University Clinics Brussels, Avenue Hippocrate 10, B-1200 Brussels, Belgium; 6 Department of Paediatric Haemato-Oncology, Llandough Hospital, Penlan Road, Llandough, Penarth, Vale of Glamorgan CF64 2XX, UK

**Keywords:** liver tumour, hepatic epithelioid haemangio-endothelioma, chemotherapy, ifosfamide, cisplatin, carboplatin, etoposide, adriamycin, vincristine, actinomycin D, liver transplantation

## Abstract

Hepatic epithelioid haemangio-endothelioma (HEHE) is an endothelium-derived tumour of low-to-medium grade malignancy. It is predominantly seen in adults and is unresponsive to chemotherapy. Liver transplantation is an accepted indication when the tumour is unresectable. Hepatic epithelioid haemangio-endothelioma is very rare in children and results after transplantation are not reported. The aim of this study is to review the experience of three European centres in the management of HEHE in children. A retrospective review of all paediatric patients with HEHE managed in three European centres is presented. Five children were identified. Four had unresectable tumours. The first had successful resection followed by chemotherapy and is alive, without disease 3 years after diagnosis. One child died of sepsis and one of tumour recurrence in the graft and lungs 2 and 5 months, respectively, after transplant. Two children who had progressive disease with ifosfamide-based chemotherapy have had a reduction in clinical symptoms and stabilisation of disease up to 18 and 24 months after the use of platinum-based chemotherapy. HEHE seems more aggressive in children than reported in adults and the curative role of transplantation must be questioned. Ifosfamide-based chemotherapy was not effective. Further studies are necessary to confirm if HEHE progression in children may be influenced by platinum-based chemotherapy.

Hepatic epithelioid haemangio-endothelioma (HEHE) is a rare tumour of vascular origin. Hepatic epithelioid haemangio-endothelioma is a tumour of low-to-intermediate grade malignancy and is distinct from the benign tumour ‘infantile haemangio-endothelioma’ and the malignant tumour ‘angiosarcoma’. Although the usual clinical course is characterised by both a very slow progression and a malignant behaviour with local recurrence and distant metastasis, its natural behaviour has also been described as unpredictable (Dietze *et al*, 1989; Makhlouf *et al*, 1999). Prolonged survival without treatment (Läuffer *et al*, 1996; Makhlouf *et al*, 1999; Uchimura *et al*, 2001) and even spontaneous clearance has been seen, while in others the tumour has progressed rapidly.

In the absence of effective chemotherapy strategies, tumour resection is the only management option. Many cases present at diagnosis with multifocal hepatic disease or large, widespread tumours and conventional liver surgery is not possible. Since HEHE progression is usually very slow, liver transplantation has been proposed as a reasonable option in these cases (Scoazec *et al*, 1988; Madariaga *et al*, 1995). The 5-year actuarial survival following liver transplantation for HEHE in adults is as high as 71% (disease-free survival=60%) (Madariaga *et al*, 1995).

Most reports have been in adults and a female predominance has been seen. In children, HEHE is extremely rare and management is usually as for adults; however, very little information is available in literature about the disease characteristics and outcome in paediatric age range. For the purpose of this retrospective review and report, a small series of children with HEHE was collected from four European centres.

## PATIENTS AND METHODS

Patients were collected from three European paediatric liver units (St Luc University Clinics Brussels, Belgium; Transplant Unit, Bergamo, Italy; and Liver Unit, Birmingham Children's Hospital, Birmingham, UK) and the Paediatric Haematology Oncology Unit, Llandough Hospital, Wales, UK. All children (<16 years) with histologically proven HEHE (typical histologic findings, Factor VIII-related-Ag positive cells) were included for this retrospective study. Details regarding demographic characteristics, mode of presentation, results of investigations, chemotherapy regimen, type of interventions and the outcome were recorded.

## RESULTS

Five children (four female) with a median age of 10.9 years (range 1.35–12.9 years) were identified from the participating centres. Demographic details are shown in Table 1

**Table 1:** Demographic features

**Case no.**	**Age at presentation (years)**	**Sex**	**Mode of presentation**	**Primary management**
1	4.4	Female	Abdominal mass	Resection
2	13	Female	Budd Chiari syndrome	Liver transplantation
3	1.3	Female	Budd Chiari and intractable ascites	Liver transplantation
4	12	Female	Abdominal pain	Chemotherapy
5	10	Male	Abdominal Pain	Chemotherapy

. Two children presented with Budd Chiari syndrome and one with a rapidly growing abdominal mass, and two had only vague upper abdominal pain. Liver function tests and serum alpha feto-protein levels were within the normal range for all cases at presentation. CT scan of abdomen showed a large mass in the centre of the liver in one patient (case 1), while in all the others multiple nodules involving both liver lobes were seen. One child had lung metastases at presentation (CT scan). The diagnosis was confirmed by liver biopsy and positive immunohistochemistry of tumour cells for factor VIII-related antigen.

The management strategies and outcome are summarised in Table 2

**Table 2:** Management strategy and outcome

**Case no.**	**Primary management**	**Secondary management**	**Outcome**
1	Tumour resection (*ex situ*)	Elective post-resection chemotherapy^a^	Alive and disease free, 3 years after diagnosis
2	Total hepatectomy (transplant)		CMV pneumonia, death 2 months after transplant
3	Total hepatectomy (transplant)	Recurrence at 3 months → chemotherapy^a^	Recurrence, death 5 months after transplant
4	Chemotherapy (A)^a^	Rescue chemotherapy (B)^a^	Alive, stable disease, 2 years after diagnosis
5	Chemotherapy (A)^a^	Rescue chemotherapy (B)^a^	Alive, stable disease, 18 months after diagnosis

a Chemotherapy regimens: see Table 3.

. Tumour resection was possible in only one patient; she had a huge centrohepatic tumour and, in order to achieve successful surgical resection, a total hepatectomy had to be done, followed by an *ex vivo* extended hepatectomy and by auto-transplant of the healthy residual liver segment. Macroscopically, a radical resection was achieved, but there was evidence of microscopic invasion in the residual liver. She underwent chemotherapy (Table 2) after surgery and, interestingly, she is currently alive and disease free, 3 years after surgery (28 months after cessation of chemotherapy, 8 years of age).

Two children underwent liver transplantation. Both presented with unresectable liver mass causing Budd–Chiari-like syndrome, and were proposed for liver transplantation as primary management. One of the two died 2 months after transplantation, from respiratory failure due to lung fibrosis secondary to cytomegalovirus infection. The other child developed lung metastases and recurrence of tumour in the graft 3 months after transplant; she then received chemotherapy (one course) with no effect on tumour and died (5 months after transplant).

The two most recent patients were started on chemotherapy after the diagnosis. They presented with a few-month story of abdominal pain and weight loss, and a widespread liver tumour was found and, in the second case (case 5), lung metastases. In both cases, no response was seen during chemotherapy (case 4-A and 5-A in Table 3

**Table 3:** Chemotherapy and outcome

**Case no.**		Treatment (see details*)	**Response to treatment**
1		SIOP MMT 952 protocol	Unable to assess response (microscopic residues), no clinical recurrence after cessation of chemotherapy
2		No chemotherapy	Refractory disease and death
3		SIOP MMT 952 protocol	No response
4	A	SIOP MMT 953 B protocol	Developed pulmonary metastasis during treatment (7 weeks) → rescue chemotherapy (B)
	B	SIOPEL 3 High Risk	Stable disease after 4 cycles of treatment
5	A	SIOP MMT 952 protocol	Progressive disease after 2 courses → rescue chemotherapy (B)
	B	Carboplatin, etoposide	Stable disease after six cycles of treatment

**Figure 1:**
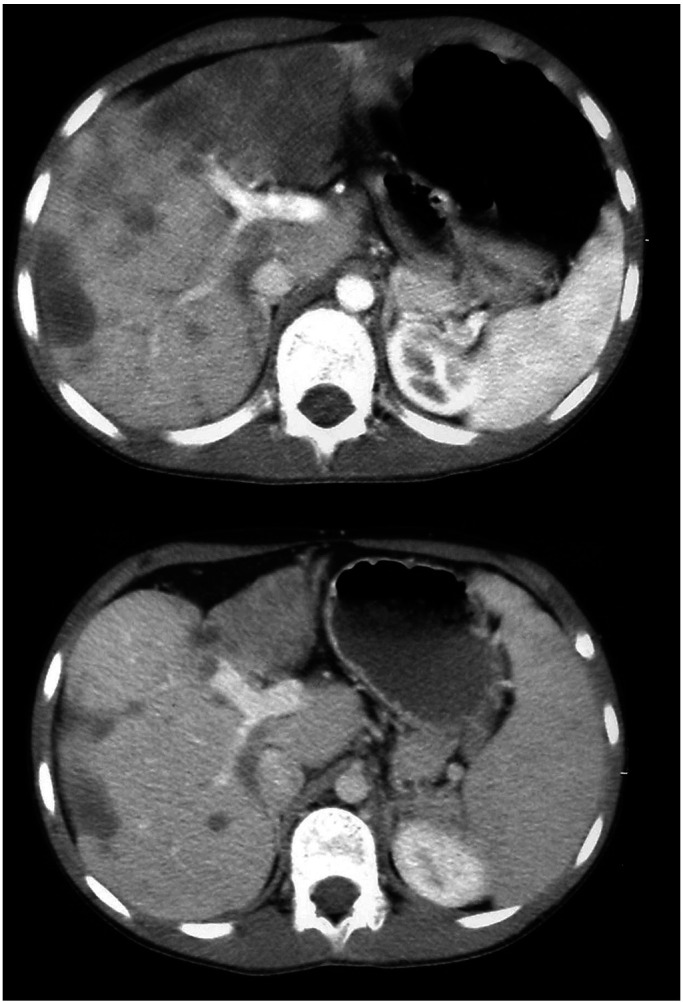
Hepatic epitheloid haemangio-endothelioma in a 10-year-old child. CT scan aspect before and after chemotherapy (carboplatin, etoposide), showing some response to chemotherapy that was associated with resolution of clinical symptoms.

* SIOP MMT 952 protocol: vincristine 1.5 mg m^−2^ (weeks 1, 2, 3, 4, 5, 6, 7, 10, 13, 16, 19, 22 and 25); actinomycin D 1.5 mg m^−2^ (weeks 1, 4, 7, 10, 13, 16, 19, 22 and 25); ifosfamide 6 g m^−2^ – over 2 days (weeks 1, 4, 7, 10, 13, 16, 19, 22 and 25). ^*^SIOP MMT 953 B protocol: vincristine 1.5 mg m^−2^ (weeks 1, 2, 3, 4, 5, 6, 7, 10, 13, 16, 19, 22 and 25); actinomycin D 1.5 mg m^−2^ (weeks 1, 10 and 19); ifosfamide 9 g m^−2^ over 3 days (weeks 1, 7, 10, 16, 19 and 25); carboplatin 500 mg m^−2^ (weeks 4, 10 and 22); epirubicin 150 mg m^−2^ (weeks 4, 10 and 22); etoposide 150 mg m^−2^ over 3 days, (weeks 7, 16 and 25). ^*^SIOPEL 3 high risk (protocol for hepatoblastoma): cisplatin 80 mg m^−2^ (days 1, 29, 57 and 85); carboplatin 500 mg m^−2^ (days 15, 43 and 71); doxorubicin 60 mg m^−2^ over 48 h (days 15, 43 and 71). ^*^Carboplatin, etoposide: carboplatin 600–800 mg m^−2^ per course; etoposide 150 mg m^−2^ (days 1, 2 and 3 each course).

), with a slight increase of tumour masses and development of lung metastases in case 4. Both were then switched to a different chemotherapy regimen (4-B and 5-B in Table 3). Further imaging (CT scan) showed, in case 4, a slight reduction of the liver tumour mass and absence of progression of lung metastases; this was associated with clinical improvement and clearance of abdominal tenderness. A repeat surgical liver biopsy was done at the end of chemotherapy where active HEHE was still found; interestingly, she is off chemotherapy for 2 years, clinically well and without evidence of further disease progression. The other child (case 5) had lung metastases at diagnosis that grow during the initial chemotherapy; however, there was a reduction of primary tumour size and no further progression of lung nodes after chemotherapy had been changed (Table 3). The latter child is well 16 months after diagnosis and his clinical symptoms have regressed, but he has now moderate portal hypertension. The tumour sites did not progress further, but he has been off chemotherapy for only few months and he waits further re-evaluation of tumour extension (Figure 1

).

## DISCUSSION

Hepatic epithelioid haemangio-endothelioma is a rare and unique neoplasm of vascular origin, now considered a entity distinct from usual haemangioma, infantile type of haemangio-endothelioma or angiosarcoma (Ishak *et al*, 1984). Hepatic epithelioid haemangio-endothelioma is generally described as a low-to-intermediate grade malignant tumour (Ishak *et al*, 1984; Dietze *et al*, 1989; Läuffer *et al*, 1996). It seems to be more frequent among young women and the role of prolonged use of oral contraceptives has been questioned (Makhlouf *et al*, 1999). It may be found incidentally but around half of patients present with upper abdominal pain or discomfort; rare presentations include jaundice, Budd–Chiari-like syndrome or liver failure. Radiological features (CT or MRI) vary: HEHE may present as a large liver mass (one case in this series), but more often the tumour is diffuse with multiple peripheral hepatic lesions with capsular retraction, peripheral contrast enhancement and evidence of multiple calcifications (Van Beers *et al*, 1992). The latter lesion type has been considered as a progression, with time, of the former unifocal mass type. Diagnosis can only be confirmed with histology (preferably a surgical biopsy) and the finding of factor VIII-related-Ag positive cells on immunohistochemical staining (Ishak *et al*, 1984; Uchimura *et al*, 2001).

Since Ishak *et al* reported first series of 32 cases in 1984, fewer than 200 cases have been reported in literature. Makhlouf *et al* (1999) reported the largest clinicopathologic study of 137 cases of various age ranging from 12 to 86 years (including 32 reported by Ishak *et al*, 1984). Out of this series, only seven cases were less than 20 years of age, and their treatment and outcome was not analysed separately. Overall, there is a lack of information in literature about HEHE in children and it is not known whether the behaviour of HEHE in children is similar to that of adults.

In adults, HEHE is characterised by a very slow progression (Demetris *et al*, 1997), and possible prolonged survival without treatment (Makhlouf *et al*, 1999). Only a few cases can be managed by partial liver resection, since the tumour tends to be widespread within liver at the time of diagnosis. In that setting, liver transplantation has been proposed with reasonable success since the disease usually progresses extremely slowly, even extra-hepatic disease has not been considered a contraindication (Scoazec *et al*, 1988; Kelleher *et al*, 1989; Madariaga *et al*, 1995; Ben-Haim *et al*, 1999; O’Grady, 2000). Madariaga *et al* (1995) reported a 71% 5-year actuarial patients survival (60% actuarial disease-free survival at 5 years post-OLT). Surprisingly, prolonged disease-free survival has been seen in transplanted patients who had disseminated disease, when other patients transplanted with disease confined to the liver developed rapid recurrence and metastasis (Demetris *et al*, 1997; Ben-Haim *et al*, 1999).

Report of children after transplantation is anecdotical; Taege *et al* (1999) reported one child who had a slowly progressing HEHE despite chemotherapy (no extra-hepatic disease) and who benefited from transplantation. He is alive and well free of disease 3 years after transplantation (Dr Melker, Hannover – personal communication). In the current series, one child who survived the post-transplant period developed rapid disease recurrence and died; the latter pattern of disease progression is more similar to that reported after transplantation for angiosarcoma. Interestingly, the child reported by Taege *et al* (1999) was older and did receive pre-operative chemotherapy, while the child in this series was younger and did not receive pre-operative chemotherapy.

It is usually considered that chemotherapy is not effective against HEHE in adults, and the few paediatric reports are in line with this consideration. Dietze *et al* (1989) described a 12-year-old girl who died within 1.5 months after starting chemotherapy. In their case report, Taege *et al* (1999) considered that chemotherapy was unsuccessful and decided to propose transplantation. However, our observations suggest that some response may be observed and this would support developing further management protocols. Although the follow-up in our series is short, one child with a very rapidly growing HEHE, who had resection followed by chemotherapy, is well and free of recurrence 3 years after operation, despite microscopic residual disease after surgery. Also, tumour progression seemed to slow down and liver masses decreased in size after chemotherapy in two patients with extensive liver disease and lung metastases.

Overall, this small series and the review of literature suggest that HEHE in the paediatric population, particularly in very young children, does not appear to behave as favourably as HEHE seen in adults. On the contrary, it is often a relatively rapidly growing tumour with a more malignant behaviour. If resection of the tumour is possible, this is the first choice of treatment in adults and children. However, if this is not possible, transplantation may not be the best management option for children and chemotherapy as pre-operative treatment or as the only treatment should be considered. It appears both from this report and that of Dietze *et al* (1989) that ifosfamide-based chemotherapy is not effective. However, three out of four patients in our series who had chemotherapy did improve and remain alive, including two who were deteriorating rapidly prior to treatment. Chemotherapy with carboplatin, cisplatin and adriamycin stabilised the disease in one patient and carboplatin and etoposide brought about a partial response followed by stabilised disease in another. Moreover, Pinet *et al* (1999) have reported a complete response after carboplatin and etoposide in an aggressive form of pleural epithelioid haemangio-endothelioma. Overall, we suggest that carboplatin and etoposide be considered for further study in paediatric HEHE. One case report suggests interferon alpha may also be effective (Kayler *et al*, 2002).

In conclusion, HEHE in children may have a more malignant behaviour compared to that reported in adults and results of surgery alone are not good. Liver transplantation for cases with unresectable disease, as done in adults, may therefore not be appropriate for managing children with HEHE. We believe that new chemotherapy or alternative drug regimens should be explored as primary management of nonresectable HEHE tumours. It is possible that more effective chemotherapy may, in turn, increase the chance of offering surgical management as a complementary treatment and with improved outcome; in that context also, the place of transplantation for children with HEHE may be reconsidered in the future.
